# Supplementary material for: Prevention of DNA Replication Stress by CHK1 Leads to Chemoresistance Despite a DNA Repair Defect in Homologous Recombination in Breast Cancer
Source: Cells. 2020 Jan 17;9(1):238. doi: 10.3390/cells9010238 (PMC7017274; doi:10.3390/cells9010238)
Supplement: Supplementary file 1 [file cells-09-00238-s001.pdf]

Prevention of DNA replication stress leads to chemoresistance despite a DNA repair defect in homologous recombination in TNBCs

Felix Meyer, Saskia Becker , Sandra Classen, Ann Christin Parplys, Wael Yassin Mansour, Britta Riepen, Sara Timm, Claudia Elisabeth Rübe, Maria Jasin, Harriet Wikman, Cordula Petersen, Kai Rothkamm, Kerstin Borgmann

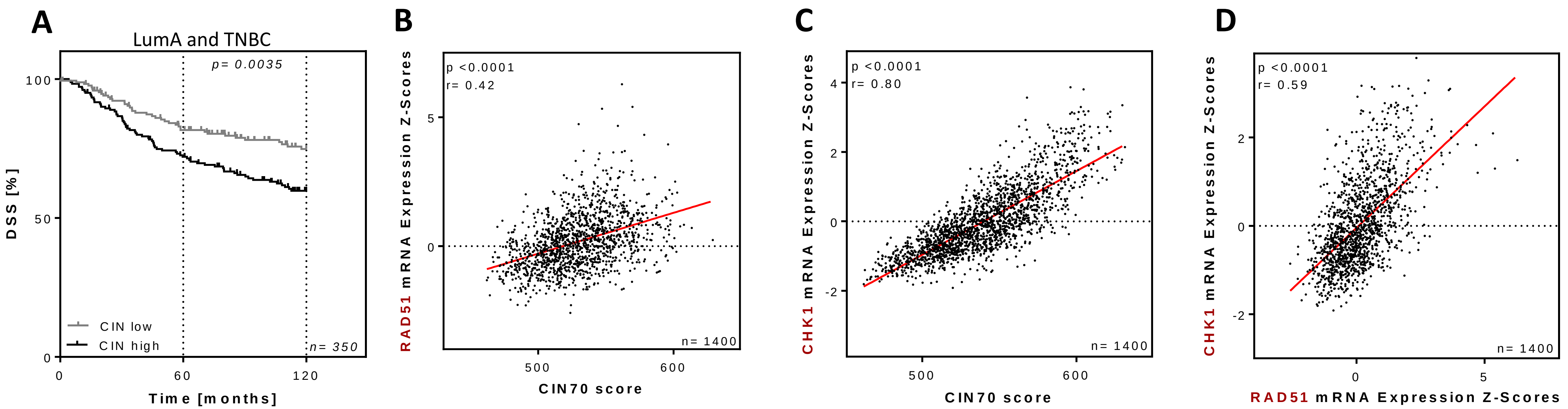

Fig. S1: (A) Kaplan-Meier survival analysis of the CIN70 score as prognostic factor for DSS in 350 patients with luminal A or Triple-negative breast cancer (log-rank test). DSS is plotted against time. (B, C) Correlation of the CIN70 score and the mRNA expression of RAD51 and CHK1. The mRNA expression values for RAD51 (B) or CHK1 (C) were sorted by magnitude, plotted against the corresponding CIN70 scores and correlated. (D) Correlation of CHK1 and RAD51 mRNA expression. The mRNA expression values of CHK1 were sorted by magnitude and plotted against the corresponding RAD51 mRNA-expression values. For statistical analysis the Pearson coefficient and p values were calculated.

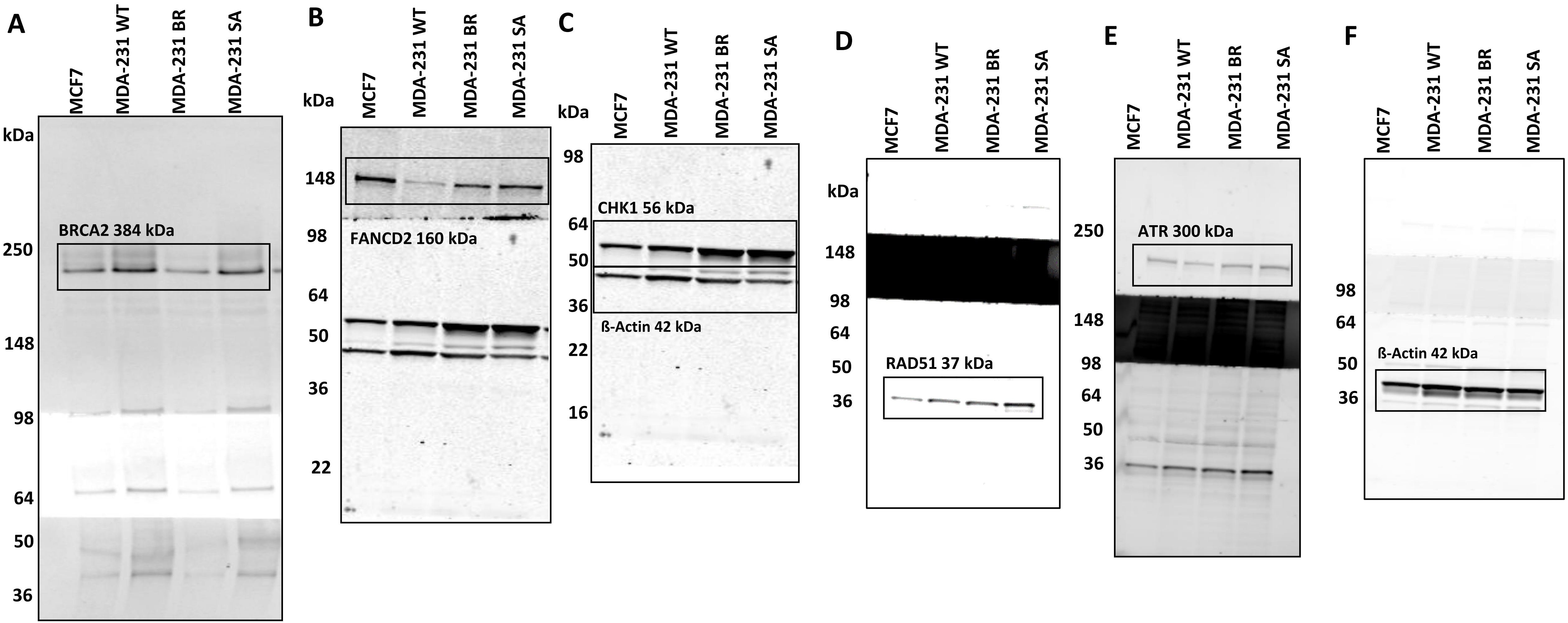

Fig. S2A: The full length blots of Figure 2A. Crop lines are indicated in the full length western blots for BRCA2 (A), FANCD2 (B), CHK1 (C), RAD51 (D), ATR(E) and ß-actin(F). After reaction with the BRCA2-antibody the same blot was stripped, re-blocked with skim milk and then reacted with the anti-FANCD2, anti-CHK1 and anti-ß-actin antibodies. kDa: Kilodalton. Only black framed bands were used in figure 1A.

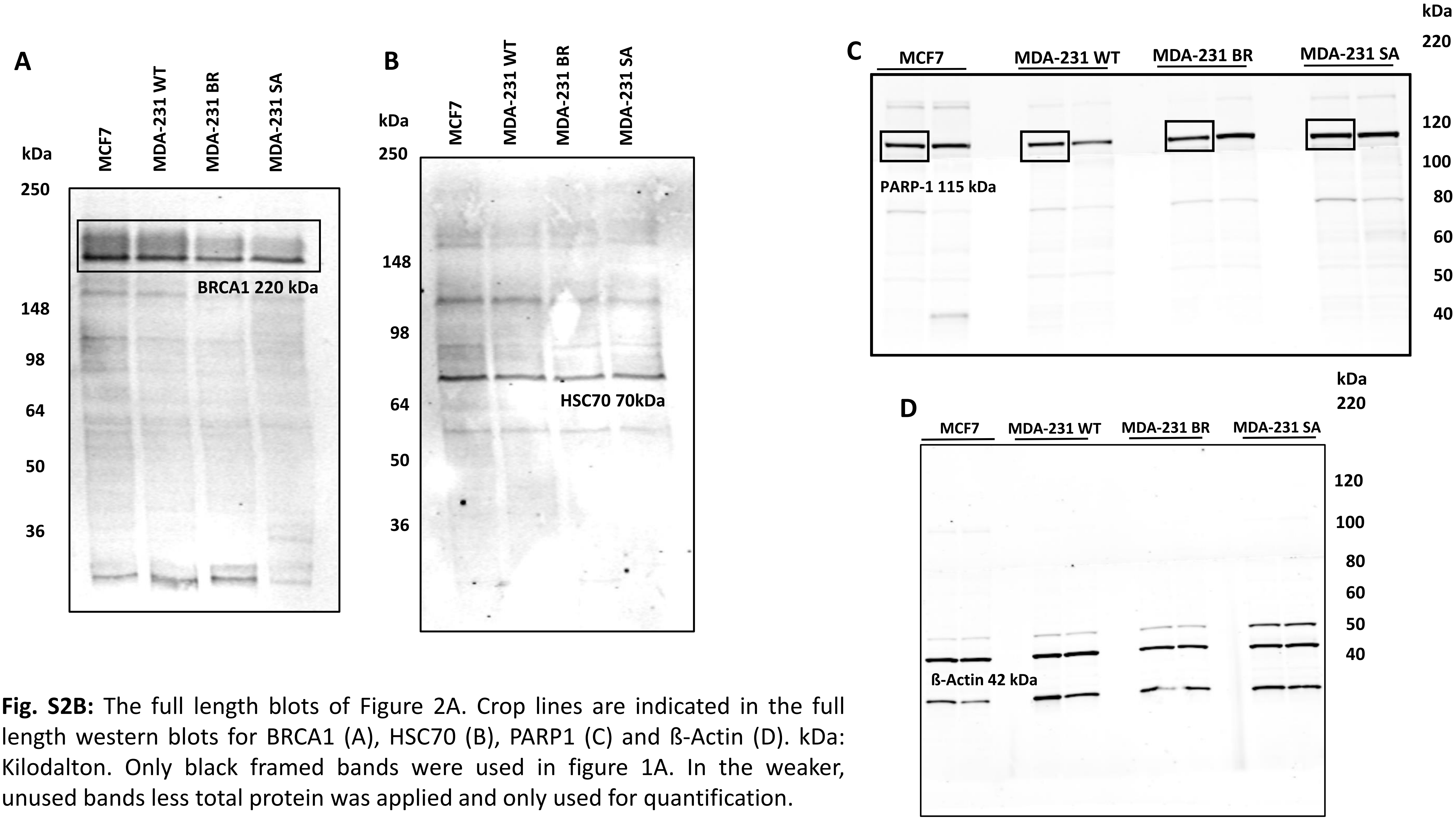

Fig. S2B: The full length blots of Figure 2A. Crop lines are indicated in the full length western blots for BRCA1 (A), HSC70 (B), PARP1 (C) and ß-Actin (D). kDa: Kilodalton. Only black framed bands were used in figure 1A. In the weaker, unused bands less total protein was applied and only used for quantification.

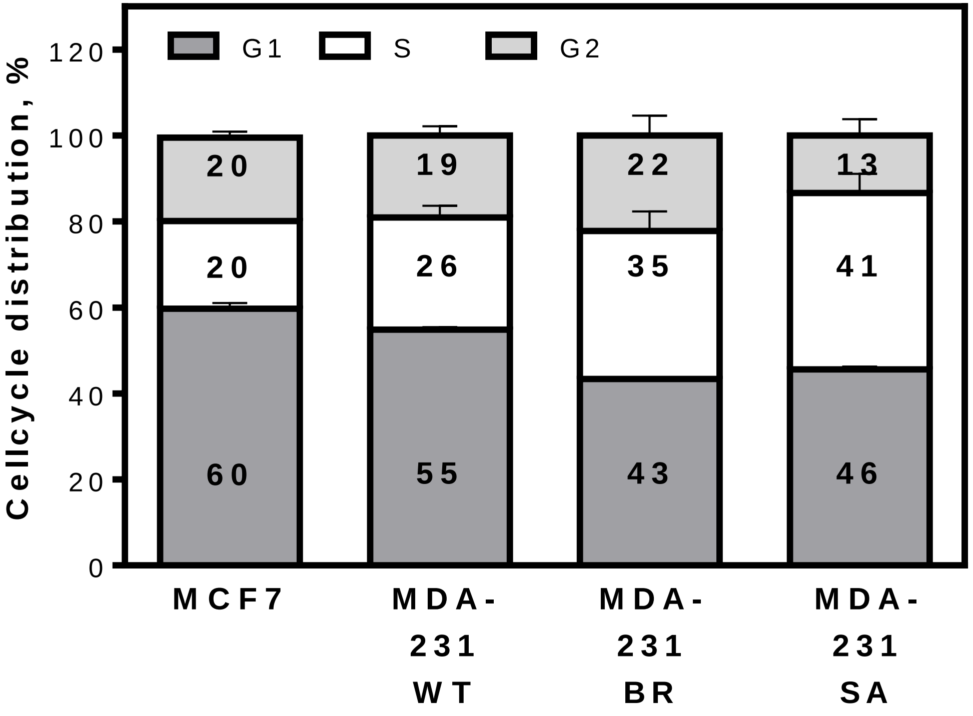

Fig S3: Cell cycle distribution from the luminal and the TNBC cell lines. Exponentially growing cells were fixed, the DNA was labelled with propidium iodide and the cell cycle phases were determined by analysis of the DNA content in the flow cytometer. Shown are means from three independent experiments ± SEM.

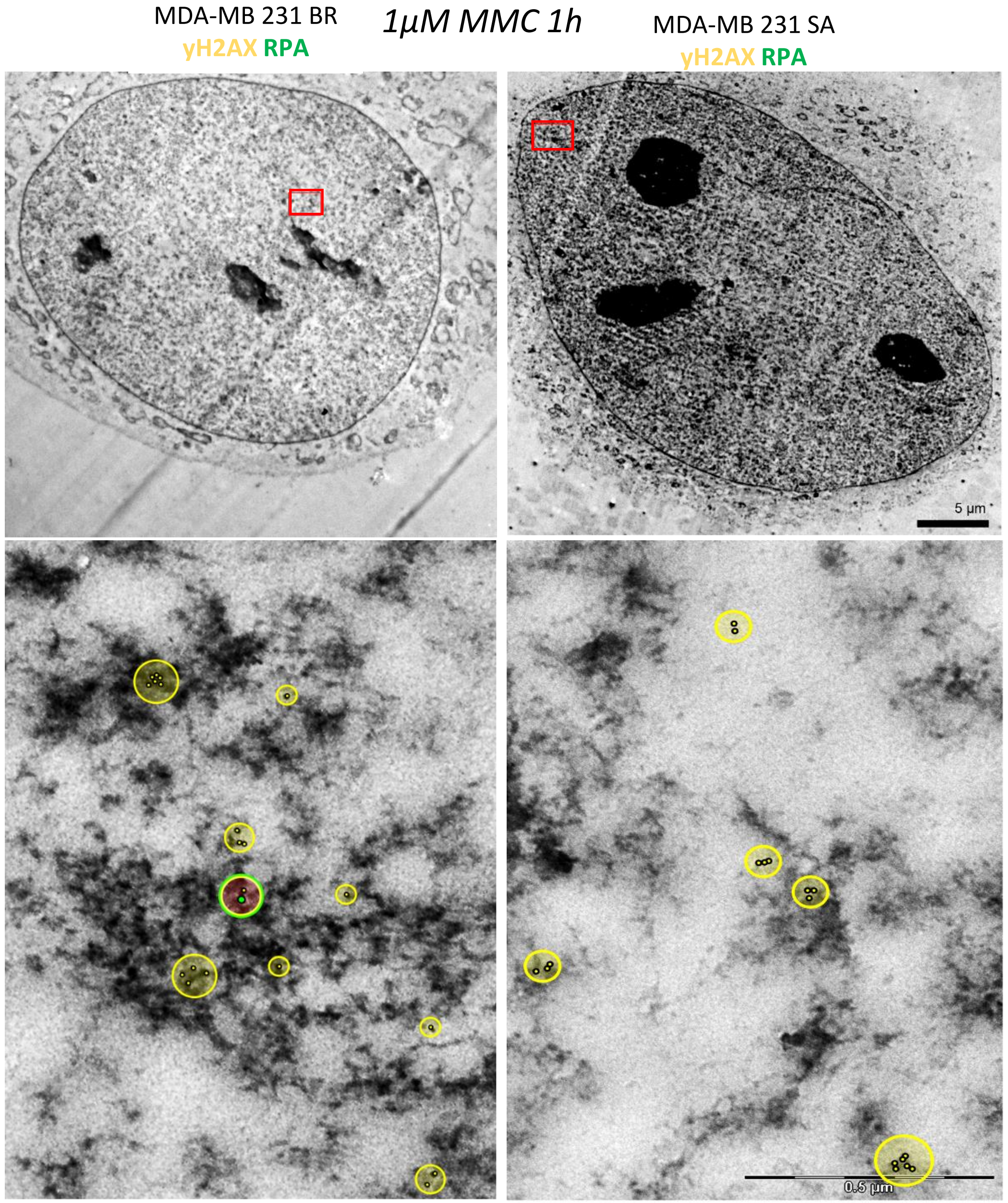

Fig. S4: Transmission electron microscopy shows colocalization of gold-labeled yH2AX (yellow) and RPA (green) for MDA-MB-231 BR and MDA-MB-231 SA cells 24h after treatment with 0.5μg/mL MMC for 1h within nuclear ultrastructure mainly associated to heterochromatic regions.

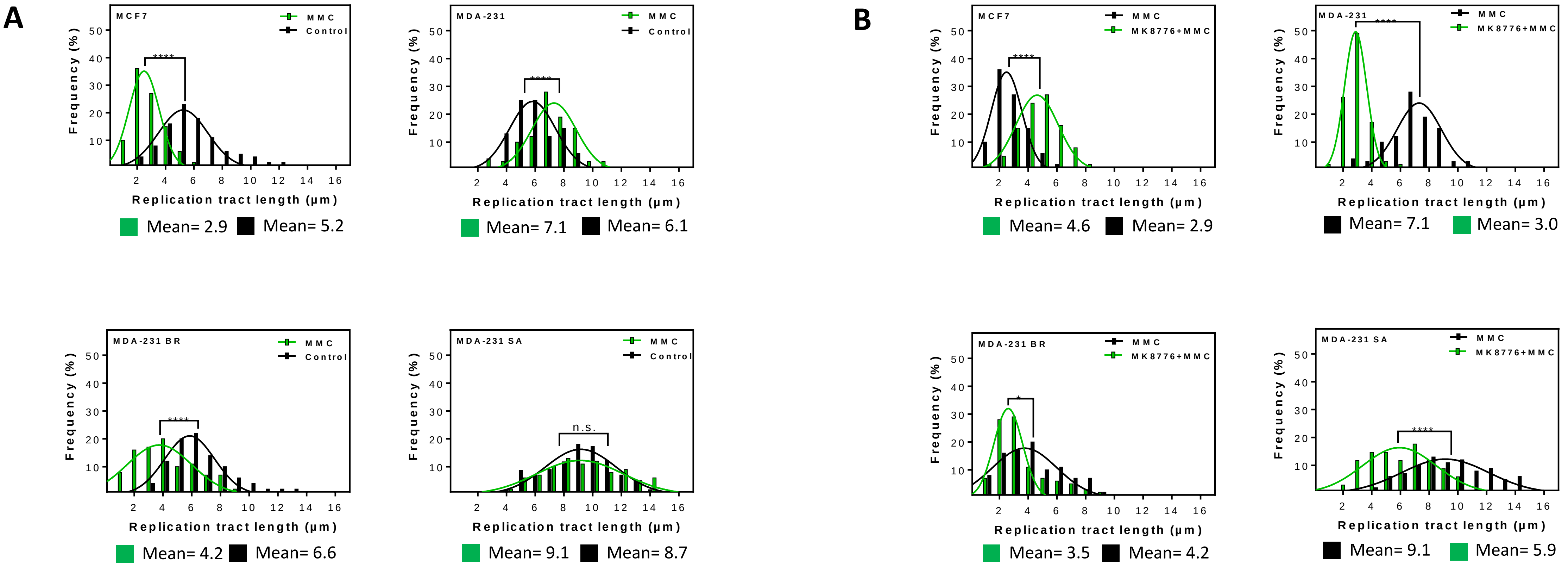

Fig. S5: CHK1 inhibition induces replication stress only in MMC resistant cells. (**A**) Frequency distribution of DNA fiber lengths (IdU) in untreated and MMC treated cells. Exponentially growing cells were sequentially labeled with CldU and IdU in the absence or presence of mitomycin C (0.1µM). DNA was spreaded and incorporated CldU and IdU was detected with appropriate antibodies. Shown are means ± SEM of DNA fiber lengths (IdU) frequency distributions of three independent experiments. Asterisks (*) represents significant differences (** $p < 0.01$; *** $p < 0.0001$, Student's t-test). (**B**) DNA fiber lengths of IdU labeled tracts after treatment with mitomycin C in the presence of the CHK1 inhibitor MK8776 (1µM). Exponentially growing cells were incubated for 2h with MK8776 and sequentially labeled with CldU and IdU (plus 0.1µM MMC), DNA was spreaded and incorporated nucleotides were detected with the appropriate antibodies. The frequency distribution of DNA fiber lengths in the first label (CldU) of three independent experiments is shown. Asterics (*) represents significant differences (*p<0.05; **p<0.01; ****p<0.0001, Student´s t-test).

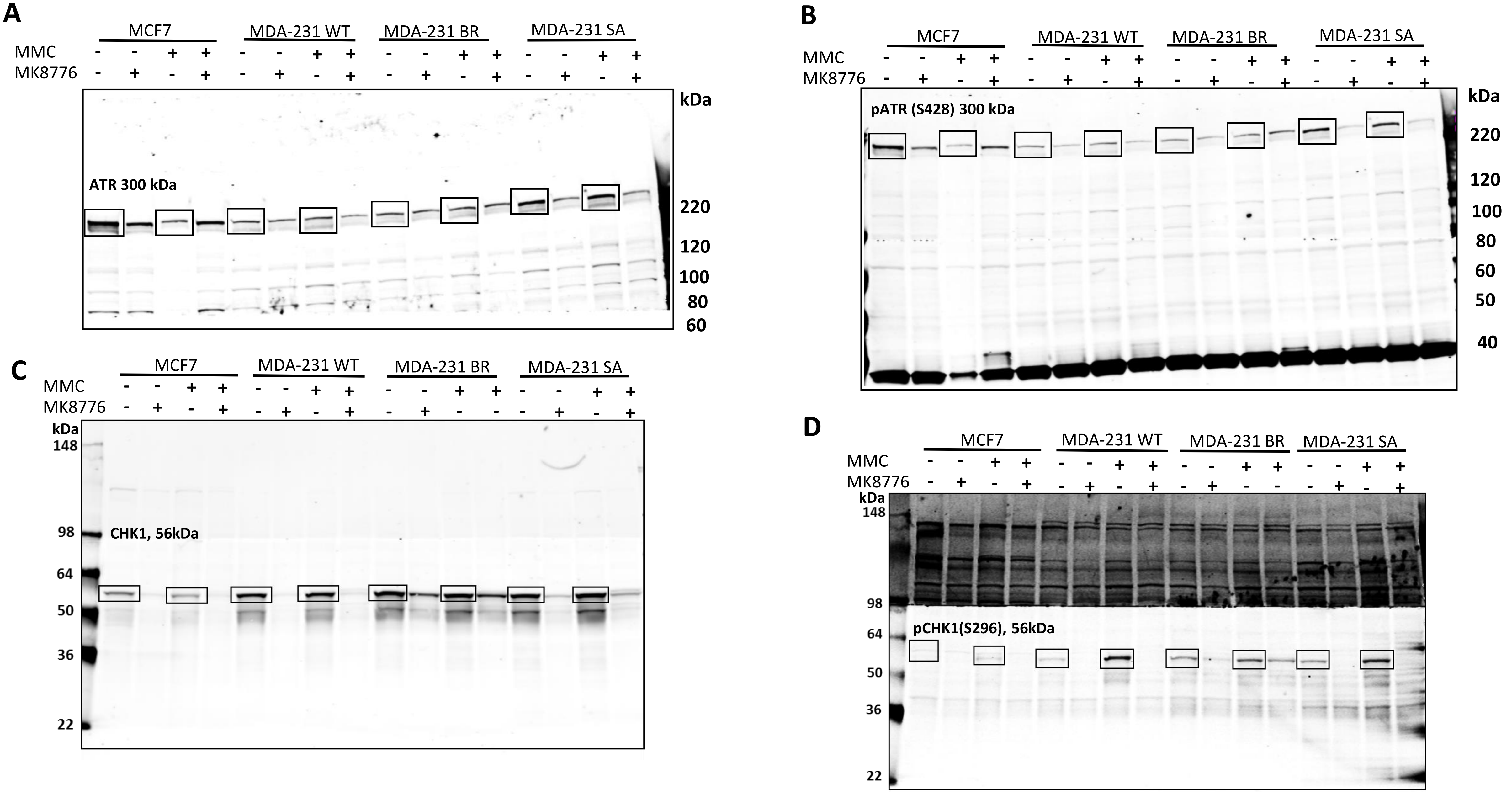

Fig. S6A: The full length blots of Figure 4B. Crop lines are indicated in the full length western blots for (A) ATR, (B) pATR, CHK1 (C) and pCHK1 (D). kDa: Kilodalton. Only black framed bands were used in figure 4A. The weaker, unused bands are the same cells, but additionally treated with MK8776.

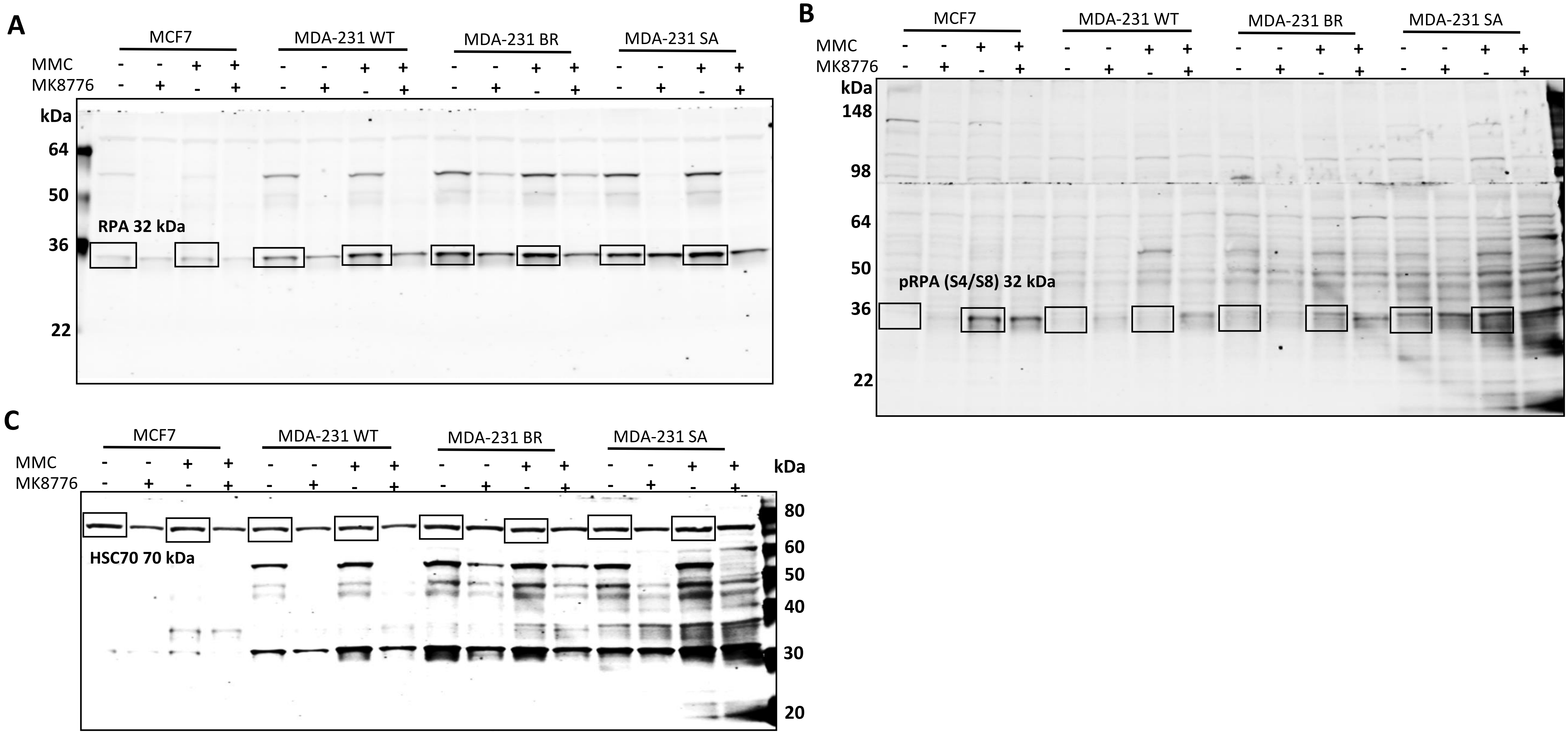

Fig. S6B: The full length blots of Figure 4B. Crop lines are indicated in the full length western blots for (A) RPA, (B) pRPA and (C) HSC70. kDa: Kilodalton. Only black framed bands were used in figure 4A. The weaker, unused bands are the same cells, but additionally treated with MK8776.

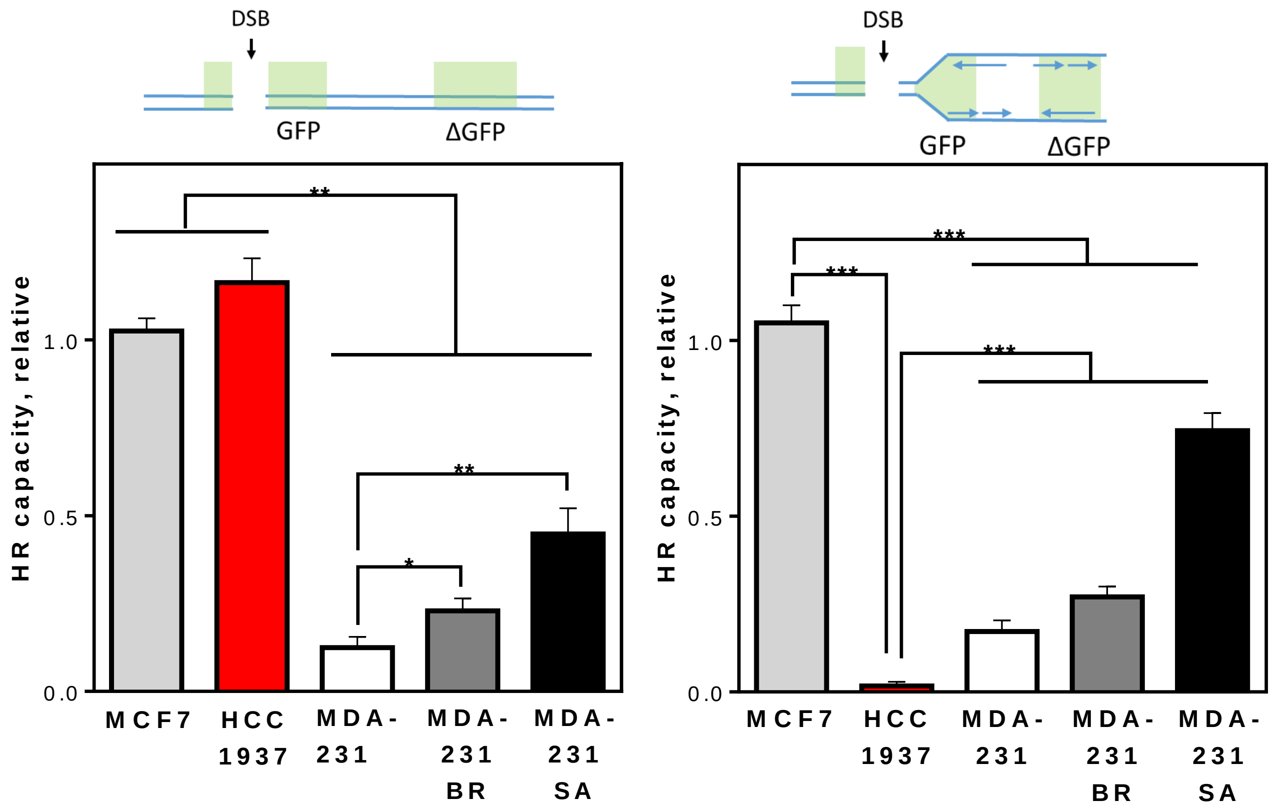

Fig. S7: Repair of frank and (left) replication-associated DSB (right), measured by plasmid reconstruction assay and analyzed by FACS. Cells were transiently transfected with DR-GFP construct (B) or DR-ori-GFP plus the ori-activating MSCV-N EBNA1 construct (B) for 24h. The number of GFP-expressing cells was normalized to the absolute HR capacity of MCF7
